# Insoluble Polymers in Solid Dispersions for Improving Bioavailability of Poorly Water-Soluble Drugs

**DOI:** 10.3390/polym12081679

**Published:** 2020-07-28

**Authors:** Thao T.D. Tran, Phuong H.L. Tran

**Affiliations:** 1 Institute of Research and Development, Duy Tan University, Danang 550000, Vietnam; trantdinhthao@duytan.edu.vn; 2 The Faculty of Pharmacy, Duy Tan University, Danang 550000, Vietnam; 3 Deakin University, School of Medicine, IMPACT, Institute for Innovation in Physical and Mental Health and Clinical Translation, Geelong, Australia

**Keywords:** solid dispersion, controlled release, nano-sized solid dispersion, dissolution enhancement, insoluble carrier

## Abstract

In recent decades, solid dispersions have been demonstrated as an effective approach for improving the bioavailability of poorly water-soluble drugs, as have solid dispersion techniques that include the application of nanotechnology. Many studies have reported on the ability to change drug crystallinity and molecular interactions to enhance the dissolution rate of solid dispersions using hydrophilic carriers. However, numerous studies have indicated that insoluble carriers are also promising excipients in solid dispersions. In this report, an overview of solid dispersion strategies involving insoluble carriers has been provided. In addition to the role of solubility and dissolution enhancement, the perspectives of the use of these polymers in controlled release solid dispersions have been classified and discussed. Moreover, the compatibility between methods and carriers and between drug and carrier is mentioned. In general, this report on solid dispersions using insoluble carriers could provide a specific approach and/or a selection of these polymers for further formulation development and clinical applications.

## 1. Introduction

In the last few decades, solid dispersions (SDs) have been involved in the development of the majority of new drugs to improve dissolution rates and controlled release because these newly discovered drugs are poorly water-soluble [1,2,3,4,5]. The limited solubility of these drugs could lead to low oral availability, potential toxicity, low half-lives, and difficult formulations [6,7,8,9]. To achieve high absorption, drugs need to be dissolved in the gastrointestinal tract [10,11]. Therefore, poorly soluble drugs often result in low absorption and oral bioavailability [10,12,13,14,15,16,17].

Recent studies have shown that hydrophilic polymers, such as hydroxypropyl methylcellulose, polyvinylpyrrolidone, hydroxypropyl cellulose, and polyethylene glycol, are commonly used in the formation of SDs [18,19,20,21,22,23,24,25,26,27,28,29,30]. Moreover, ternary SDs of these polymers have also been utilized to further improve drug bioavailability [31,32,33,34,35,36,37,38,39,40,41].

**Figure 1:**
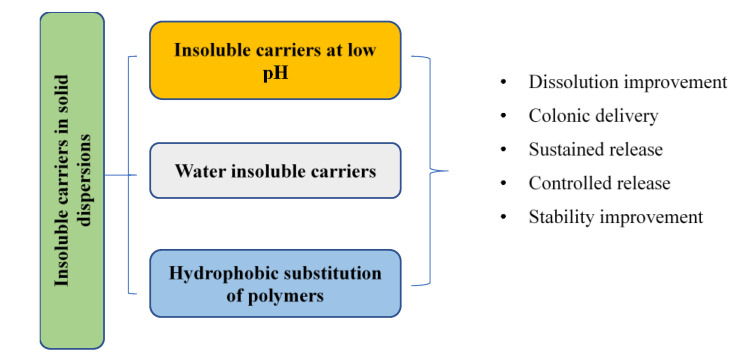
General approaches and key applications for using insoluble carriers in solid dispersions (SDs).

In addition, insoluble carriers have also been exploited in many formulations of SDs. By taking advantage of hydrophobic interactions between polymers and poorly water-soluble drugs, the polymer may easily change the drug crystallinity into an amorphous state via molecular interactions, enhancing dissolution [42,43,44,45,46,47,48]. Moreover, the insoluble property of carriers in various dissolution media might be applied to overcome the low bioavailability of drugs with pH-independent solubility. The poor solubility of certain polymers can also be utilized in the development of controlled-release SDs or even nano-sized SDs. This review, therefore, provides insight strategies for using insoluble carriers in SDs to improve drug dissolution and bioavailability. Figure 1 describes general approaches and key applications for using insoluble carriers in SDs.

## 2. Fundamental Properties and Physicochemical Characterization of SDs

Generally, SDs can be defined as the dispersion of a poorly water-soluble drug(s) in a carrier or a mixture carrier [49]. A crystalline drug can be transformed into the amorphous form once it is dispersed in polymers [50,51]. In addition, various advantages of SDs, including wettability improvement, reduced size, and porosity of particles, contribute to the dissolution enhancement and bioavailability of poorly water-soluble drugs [52]. In certain circumstances, the molecular interaction has a crucial role in the formation of the amorphous forms of drugs and maintains the stability of SDs.

However, physical stability may prevent further development of SD products. Under storage conditions, an amorphous drug in an SD can recrystallize under thermodynamic and moisture conditions [53]. Fortunately, more homogeneous amorphous SDs lead to the longer physical stability of formulations [54]. Compared to hydrophilic polymers, better hydrophobic interactions between an insoluble carrier and poorly water-soluble drugs probably result in more homogenous SDs. Moreover, hydrophobic carriers might also help to prevent moisture adsorption. Therefore, the use of insoluble carriers may be a good strategy to improve the long-term stability of SDs. With regard to chemical stability, both hydrophilic and hydrophobic compounds can protect the dispersed molecules in SDs at a certain level (e.g., against oxidative degradation) [55]. However, drug molecules, which are susceptible to pH in the gastrointestinal tract, can be degraded if an SD is fabricated from hydrophilic polymers. In contrast, insoluble carriers appear useful in chemical stability by preventing drug release in the medium, which can degrade the model drug [56].

Physicochemical characterization of SDs is an important factor in determining successful formulations and the mechanism of drug release. First, the level of drug crystallinity in SDs can be evaluated via techniques such as powder X-ray diffraction and different scanning calorimetry [57]. Second, the molecular interactions can be characterized by infrared spectroscopy, Raman spectroscopy, nuclear magnetic resonance spectroscopy, X-ray photoelectron spectroscopy, molecular modeling, quantum chemical calculation, and water vapor sorption [58,59]. Third, scanning electron microscopy, atomic force microscopy, and transmission electron microscopy are usually utilized to observe the morphologies of SDs and nano-sized particles [26,58]. The details of those methods can be found in prominent reviews [57,58,59].

## 3. Insoluble Carriers at Low pH Levels in SDs

### 3.1. Dissolution Improvement in Alkaline Environments and for Colonic Delivery

**Figure 2:**
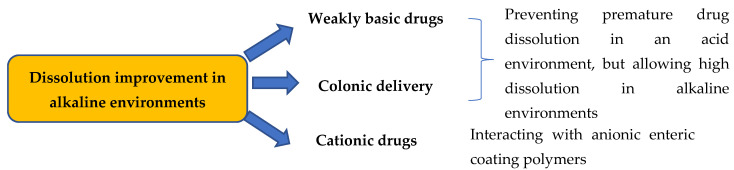
The ability to incorporate poorly water-soluble drugs in SDs using insoluble carriers at low pH levels.

Although the use of enteric coating materials as a carrier in SDs can inhibit drug release at low pH levels, an improvement in drug dissolution in neutral and alkaline media can be obtained with amorphous SDs. Figure 2 describes the ability to incorporate poorly water-soluble drugs in SDs using insoluble carriers at low pH levels. For instance, Eudragit^®^ S, which dissolves above a pH of 7, successfully changes dipyndamole to an amorphous state in an SD by the solvent method [60]. Dipyndamole is a weakly basic drug with a pH-dependent solubility that causes incomplete absorption in the gastrointestinal tract [60]. Therefore, amorphous SDs of dipyndamole significantly increases drug dissolution at pH levels above 7 to improve drug bioavailability [60]. This study also confirmed that dipyndamole and Eudragit^®^ S interact via hydrogen bonding of carboxylic groups and nitrogen atoms [60]. Similarly, Eudragit^®^ S100 has also been shown to be a promising material in SDs of berberine hydrochloride for colonic delivery [61]. In vitro cytotoxicity tests in human colon cancer cells (HCT116 and SW480) have suggested antitumor activity enhancement, which indicates the potential application of SDs with Eudragit^®^ S100 for colon cancer therapy [61].

The utilization of enteric coating agents in SDs is a potential application for weakly basic drugs with pH-dependent solubility [62,63]. For example, itraconazole is soluble in gastric fluid, but it is likely precipitated after entering the small intestines due to the pH change, leading to low bioavailability [63]. To prevent soluble drugs from dissolving in the stomach, enteric coating agents are used in SDs [62,63]. Moreover, amorphous SDs can increase drug absorption in the small intestine, where higher absorption is observed compared to the stomach [63,64,65]. Overhoff et al. successfully used hydroxypropylmethylcellulose phthalate in SDs containing itraconazole with the mentioned purposes [63]. In this study, amorphous SDs were fabricated by the solvent method using ultra-rapid freezing [63].

Anionic enteric coating polymers, such as Eudragit^®^ L100 and Eudragit^®^ L100-55, can be applied for delivering cationic drugs in SDs [66]. Maniruzzaman et al. indicated that the amide groups of propranolol HCl and diphenhydramine HCl molecularly interact with the carboxyl group of polymers via hydrogen bonding, resulting in the formation of amorphous drugs in SDs by hot-melt extrusion [66]. In this study, hot-melted extrusion played an important role due to its facilitative ability to enhance the interaction between drugs and polymers in SDs, increasing drug solubility [66,67,68,69]. However, preparations of SDs with Eudragit^®^ L100-55 should carefully take into account the temperature during the process because the resulting SDs could be degraded at temperatures below 180 °C [70,71].

### 3.2. Effects of Enteric Coating Polymers and Preparation Methods on Amorphous SDs

**Figure 3:**
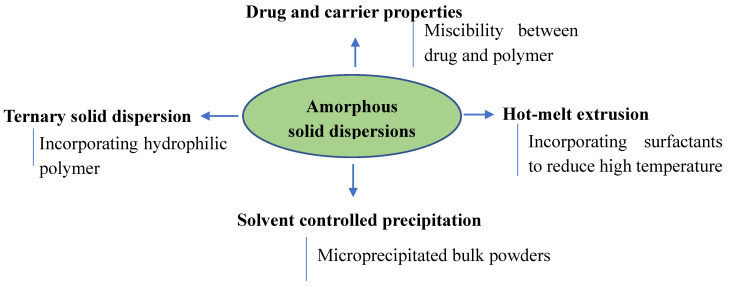
Illustration of factors from enteric coating polymers affecting amorphous SDs.

The formation of amorphous SDs with enteric coating materials depends on the drug properties, the type of carrier, and the preparation methods. Figure 3 illustrates factors from enteric coating polymers affecting amorphous SDs. Indeed, in an investigation by Hasegawa et al., the effects of six enteric coating agents on two poorly water-soluble drugs (griseofulvin and phenytoin) were analyzed, but only hydroxypropylmethylcellulose phthalates (HP-50 and HP-55) formed amorphous SDs with these drugs [72]. Drugs were still in crystal form when Eudragit^®^ L, Eudragit^®^ S, cellulose acetate phthalate, and carboxymethyl ethoxy ethyl cellulose were used [72]. In a study on MK-0364 SDs, Sotthivirat et al. showed that hydroxypropyl methylcellulose acetate succinate was more effective than hydroxypropylmethylcellulose phthalates and Eudragit^®^ L100-55 in enhancing the dissolution of a poorly water-soluble drug [73]. Moreover, hot-melt extrusion has been demonstrated as a suitable approach for preparing SDs using hydroxypropyl methylcellulose acetate succinate [74,75]. Surfactants have been recommended to be incorporated in SDs to reduce the high temperatures during the hot-melt extrusion processes, which may cause the degradation of both drug and polymers [76].

The effectiveness of SDs developed from enteric coating polymers depends on the miscibility between drug and polymer [77]. It has been noted that excessive drug loading in SDs results in drug domains that do not interact with polymer matrixes [77]. Amorphous drugs are easily transformed into drug crystals once these domains are exposed to the dissolution medium [77]. Therefore, miscibility and drug recrystallization must be considered in SD formulations with enteric coating agents [77]. The preparation method certainly affects the miscibility in SDs [78]. In an investigation of amorphous SDs of lumefantrine, Song et al. showed that more favorable acid-base interactions were observed in SDs by spray-drying compared to hot-melt extrusion because of their exposure to the solution [78].

To improve stable, amorphous SDs, Shah et al. suggested a method to precipitate SDs (so-called solvent controlled precipitation) [79]. Eudragit^®^ L100, Eudragit^®^ L100-55, Eudragit^®^ S100, hypromellose acetate succinate, and hypromellose phthalate 50 were used as carriers in this study [79]. Briefly, a poorly water-soluble drug and a polymer were dissolved in an organic solvent, which was then precipitated into the aqueous medium [79]. Based on the insoluble properties of the enteric coating agents, the aqueous medium was maintained between pH 1 and 3 to minimize the solubility of the polymers; therefore, the drug was dispersed in the inner carrier and precipitated into microparticles [79]. The precipitates were isolated and dried to form microprecipitated bulk powder with the characteristics of an amorphous SD [79]. By using this technology, enteric coating polymers prevented nucleation, protected against moisture, and maintained supersaturation, immobilizing the amorphous drug in SDs [79]. The authors proposed that the insolubility in medium and possible ionic interactions were the results of the stabilization of the amorphous SDs [79].

Further development of an efficient screening method was also proposed by the same group for the selection of polymer type, drug loading, and solvent in the development of SDs using microprecipitated bulk powder [80]. Specifically, the authors suggested a 96-well platform composed of miniaturized co-precipitation screening (including mixing drugs and enteric polymers in organic solvents, controlled precipitation, isolation, drying, and high throughput characterization) [80]. Practically, solvent-controlled precipitation has been demonstrated as an efficient method for improving the human bioavailability of poorly water-soluble drugs [81]. Vemurafenib has been chosen to prepare the microprecipitated bulk powder, increasing the human bioavailability five-fold compared to the crystalline drug [81].

Ternary SDs are effective strategies for further enhancing the dissolution of poorly water-soluble drugs [68,82,83,84,85]. Enteric coating polymers have been investigated by this approach [86,87]. Ohyagi et al. combined hypromellose and Eudragit^®^ L 100 (or a methacrylic acid copolymer) and showed that the resulting ternary SDs had improved dissolution compared to those of single-polymer SDs [86]. Differential scanning calorimetry and solid-state NMR confirmed that the hydroxyl groups (HPMC) and carboxyl groups (enteric coating polymers) interacted to form the intermolecular interactions that led to dissolution enhancement [86]. Therefore, the authors suggested that this strategy is likely a powerful approach to create SDs for poorly water-soluble drugs [86].

### 3.3. Nano-Sized SDs from Enteric Coating Polymers

Duarte et al. developed a solvent-controlled precipitation method to produce nano-SDs of carbamazepine by using microfluidization [88]. A similar precipitation process was proposed in which the precipitates were spray-dried to form nano-SDs of approximately 100 nm [88]. The authors compared the results from these SDs with those obtained with amorphous SDs prepared by spray-drying but without precipitation process [88]. The higher dissolution rate and bioavailability of the nano-SDs demonstrated that particle size played a key role in improving the bioavailability of carbamazepine [88].

Electrospinning has also been used as an alternative approach to prepare nano-sized SDs with enteric coating polymers [89]. Balogh et al. utilized Eudragit^®^ FS 100 in SDs of poorly soluble spironolactone by electrospinning and hot-melt extrusion to produce nanofibers and amorphous SDs, respectively [89]. Both methods showed impressive dissolution enhancement at a pH of 7.4 [89]. However, drug release in the gastric fluid was higher in the case of the electrospun samples compared to the extruded SDs due to the large surface area of the nanofibers [89]. In general, Eudragit^®^ FS 100 was demonstrated to be an excellent carrier in SDs, and hot-melt extrusion was recommended for colon-targeted delivery of poorly water-soluble drugs with this polymer [89].

Although hydroxypropylmethylcellulose acetate succinate has been shown to be a promising carrier in SDs, it is difficult to process using electrospinning [90,91,92]. However, this process has been used successfully to produce nanofibers of spironolactone by adjusting the conductivity in a study by Balogh et al. [90]. This study indicated the importance of solution conductivity in electrospinning with hydroxypropylmethylcellulose acetate succinate because the drug dissolution from nanofibers was dependent on the adjusted conductivity [90]. Compared with other hydrophilic polymers (i.e., hydroxypropyl methylcellulose and polyvinylpyrrolidone K-30), hydroxypropylmethylcellulose acetate succinate is less effective in improving the dissolution of darunavir [93].

Hassouna et al. proposed a combination of the emulsification-diffusion method and freeze-drying to prepare ibuprofen-loaded Eudragit^®^ L100-55 nanoparticles, resulting in amorphous SDs [94]. The encapsulation of the drug in Eudragit^®^ L100-55 nanoparticles not only sustained drug release but also stabilized the amorphous state during storage [94].

## 4. Water-Insoluble Carriers for SDs

### 4.1. Sustained Release and Stability Improvement

**Figure 4:**
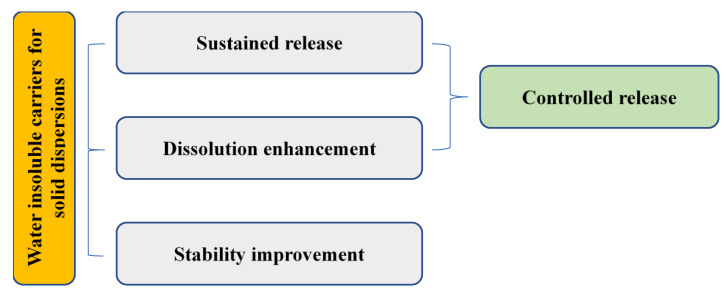
Illustration of applications of water-insoluble carriers in SDs.

Figure 4 illustrates the applications of water-insoluble carriers for SDs. For example, Eudragit^®^ RS 100 and RL 100 have been utilized in sustained-release SDs because they are insoluble at physiological pH values but can swell and become permeable to water [95,96,97]. By formulating misoprostol in SD matrices with these polymers, drug release could be slowed, and drug stability can be protected from degradation by water [96]. Interactions via hydrogen bonding and channel formation are attributed to the drug release pattern [98,99]. Compared to nanoparticles with the same Eudragit^®^ RS 100 polymer in their formulations, diclofenac sodium–Eudragit^®^ RS100 SDs are shown to have a slower drug release rate [97].

In addition to aiding in sustained drug release, Eudragit^®^ RS 100 and RL 100 are also used in a photoprotective strategy [100]. Although the SDs do not transform the drug from its crystalline form, drug release is prolonged, and the photosensitive compound diflunisal is protected due to the dispersion of the drug in the polymers in a molecular or microcrystalline form [100].

Amorphous SDs with Eudragit^®^ RS PO are developed for transdermal films because a high concentration of the released drug may enhance skin permeability [101]. However, hydrophilic excipients (e.g., gelucire, xanthan gum) are suggested to be incorporated into transdermal systems to allow water sorption and create triggered drug delivery systems [101].

### 4.2. Dissolution Improvement

Due to the low wettability of water-insoluble carriers, the amorphous SDs from these polymers have shown a lower dissolution rate compared to water-soluble polymers [102,103]. Therefore, Ngo et al. developed hydrophilic-hydrophobic polymer blends in SDs to enhance dissolution [83,104]. Specifically, zein was used as an insoluble carrier and combined with hydroxypropyl methylcellulose to modulate molecular interactions and drug crystals [83,104]. Compared to the single polymer-based SDs (zein or hydroxypropyl methylcellulose), this combination resulted in a high reduction of drug crystallinity, high wettability, and good performance of molecular interactions [104]. The use of hydrophobic polymers in SDs might facilitate molecular interactions with poorly water-soluble drugs for changing drug crystals to amorphous forms [104]. In the case of the limited dissolution rate of very poorly soluble drugs, the addition of surfactant in this polymer blend was part of a strategy to decrease drug recrystallization and increase wettability [83].

### 4.3. Controlled Release of SDs with Water-Insoluble Carriers

Under certain circumstances, dissolution enhancement and sustained release are required to improve the bioavailability of a poorly water-soluble drug. For example, Yang et al. proposed a system including two main parts: (1) SDs to improve the dissolution rate of nitrendipine and (2) the presence of Eudragit^®^ RS PO in the SDs to sustain drug release [105]. Specifically, the drug and Eudragit^®^ RS PO were dissolved in an organic solvent, which was then incorporated with Aerosil to form microspheres [105]. The drug release from the microsphere SD could be modulated by altering the amount of Eudragit RS PO [105].

In a concept similar to microsphere SDs, Huang et al. used Eudragit^®^ RL and ethylcellulose blends to control nifedipine release [106]. This study indicated that these SDs exhibited good stability because of molecularly stable interactions via hydrogen bonding between the drug and the Eudragit^®^ RL and ethylcellulose blends [106]. However, it has been noted that the internal structure of the microspheres and the physical state of nifedipine would change if high drug loading occurs in the formulations [107]. The presence of excessive amounts of drugs would form drug reservoirs, resulting in a change in the drug release kinetics [107].

## 5. Hydrophobic Substitution of Polymers in SDs

To interact with poorly water-soluble drugs, hydrophobic polymers can be substituted on polymers. This substitution would lead to the formation of a new material that can be used in SDs to enhance drug solubility and bioavailability. Orienti et al. investigated the substitution of polyvinyl alcohol with triethylene glycol monoethyl ether for preparing progesterone SDs [108]. Given the presence of the amphiphilic tetraethylene moiety in the substituted polymer, the solubility of progesterone increases with increasing polymer concentration, which improves the chance of interactions forming between the hydrophobic parts of the polymers and the drug [108].

In addition to the amphiphilicity of the new polymer, the substituted polymer concentration strongly affects the physicochemical properties of the drug. For instance, in an investigation of the effect of hydroxypropyl methylcellulose acetate succinate on the crystallization suppression of four model drugs (carbamazepine, nifedipine, mefenamic acid, and dexamethasone), a lower concentration of substituted succinoyl has resulted in a strong suppression of drug crystallization [109]. However, a high substituted succinoyl concentration has been recommended to increase the drug dissolution rate due to its high hydrophilicity [109].

In an effort to create unique material for SDs, a zein-hydroxypropylmethylcellulose conjugate has been proposed to enhance the dissolution of poorly water-soluble drugs [110,111]. In fact, the conjugates from these materials have shown amphiphilic properties and formed self-assembled nanoparticles during a dissolution test [110,111]. Therefore, the conjugate not only acts as a carrier to change drug crystallinity to an amorphous state but also encapsulates a poorly water-soluble drug in nano-size particles in dissolution media, resulting in enhanced dissolution [110,111]. Moreover, these studies have also indicated that the conjugated material is suitable for different model drugs in different gastrointestinal tract environments [110,111].

## 6. Conclusions

Substantial strategies for using insoluble carriers in SDs include pH-sensitive carriers, water-insoluble carriers, and hydrophobic substitution of polymers. In addition to improving the drug dissolution rate, these insoluble carriers in SDs could be utilized to control drug release or target delivery in the colon in the same formulations with poorly water-soluble drugs. The application of advanced nanotechnology in SDs containing insoluble carriers has shown promising approaches and materials in recent studies. Moreover, the improved stability of SDs containing insoluble carriers compared to other polymers has been reported in some cases [112]. With regard to the evaluation of SDs containing enteric coating polymers, the selection of in vitro dissolution conditions should be considered carefully. For example, one investigation demonstrated that the in vitro performance of an amorphous SD of celecoxib in fasted state simulated intestinal fluid with a pH of 7.4 was more representative of the in vivo performance of the SD than the in vitro performance in fasted state simulated intestinal fluid with a pH of 6.5 [113].

## References

[1] Bahloul B., Safta F., Lassoued M.A., Dhotel H., Seguin J., Mignet N., Sfar S. (2018). Use of mouse model in pharmacokinetic studies of poorly water soluble drugs: Application to fenofibrate. J. Drug Deliv. Sci. Technol..

[2] Göke K., Lorenz T., Repanas A., Schneider F., Steiner D., Baumann K., Bunjes H., Dietzel A., Finke J.H., Glasmacher B. (2018). Novel strategies for the formulation and processing of poorly water-soluble drugs. Eur. J. Pharm. Biopharm..

[3] Chen X.-Q., Ziemba T., Huang C., Chang M., Xu C., Qiao J.X., Wang T.C., Finlay H.J., Salvati M.E., Adam L.P. (2018). Oral Delivery of Highly Lipophilic, Poorly Water-Soluble Drugs: Self-Emulsifying Drug Delivery Systems to Improve Oral Absorption and Enable High-Dose Toxicology Studies of a Cholesteryl Ester Transfer Protein Inhibitor in Preclinical Species. J. Pharm. Sci..

[4] Azad M., Moreno J., Bilgili E., Davé R. (2016). Fast dissolution of poorly water soluble drugs from fluidized bed coated nanocomposites: Impact of carrier size. Int. J. Pharm..

[5] Tran P.H.L., Duan W., Lee B.-J., Tran T.T.D. (2019). The use of zein in the controlled release of poorly water-soluble drugs. Int. J. Pharm..

[6] Sanches B.M.A., Ferreira E.I. (2019). Is prodrug design an approach to increase water solubility?. Int. J. Pharm..

[7] Stella V.J., Nti-Addae K.W. (2007). Prodrug strategies to overcome poor water solubility. Adv. Drug Deliv. Rev..

[8] Rautio J., Kumpulainen H., Heimbach T., Oliyai R., Oh D., Järvinen T., Savolainen J. (2008). Prodrugs: Design and clinical applications. Nat. Rev. Drug Discov..

[9] Clas S.-D., Sanchez R.I., Nofsinger R. (2014). Chemistry-enabled drug delivery (prodrugs): Recent progress and challenges. Drug Discov. Today.

[10] Vithani K., Jannin V., Pouton C.W., Boyd B.J. (2019). Colloidal aspects of dispersion and digestion of self-dispersing lipid-based formulations for poorly water-soluble drugs. Adv. Drug Deliv. Rev..

[11] Kesisoglou F., Panmai S., Wu Y. (2007). Nanosizing—Oral formulation development and biopharmaceutical evaluation. Adv. Drug Deliv. Rev..

[12] Lipinski C.A., Lombardo F., Dominy B.W., Feeney P.J. (2001). Experimental and computational approaches to estimate solubility and permeability in drug discovery and development settings1PII of original article: S0169-409X(96)00423-1. The article was originally published in Advanced Drug Delivery Reviews 23 (1997) 3–25.1. Adv. Drug Deliv. Rev..

[13] Lipinski C. (2002). Poor aqueous solubility—An industry wide problem in drug discovery. Am. Pharm. Rev..

[14] Zhao J., Yang J., Xie Y. (2019). Improvement strategies for the oral bioavailability of poorly water-soluble flavonoids: An overview. Int. J. Pharm..

[15] Shi Y., Erickson B., Jayasankar A., Lu L., Marsh K., Menon R., Gao P. (2016). Assessing Supersaturation and Its Impact on In Vivo Bioavailability of a Low-Solubility Compound ABT-072 With a Dual pH, Two-Phase Dissolution Method. J. Pharm. Sci..

[16] Kumar R. (2019). Nanotechnology based approaches to enhance aqueous solubility and bioavailability of griseofulvin: A literature survey. J. Drug Deliv. Sci. Technol..

[17] Shi X., Song S., Ding Z., Fan B., Huang W., Xu T. (2019). Improving the Solubility, Dissolution, and Bioavailability of Ibrutinib by Preparing It in a Coamorphous State With Saccharin. J. Pharm. Sci..

[18] Ogawa N., Hiramatsu T., Suzuki R., Okamoto R., Shibagaki K., Fujita K., Takahashi C., Kawashima Y., Yamamoto H. (2018). Improvement in the water solubility of drugs with a solid dispersion system by spray drying and hot-melt extrusion with using the amphiphilic polyvinyl caprolactam-polyvinyl acetate-polyethylene glycol graft copolymer and d-mannitol. Eur. J. Pharm. Sci..

[19] Loh G.O.K., Tan Y.T.F., Peh K.K. (2014). Hydrophilic polymer solubilization on norfloxacin solubility in preparation of solid dispersion. Powder Technol..

[20] Riekes M.K., Kuminek G., Rauber G.S., de Campos C.E.M., Bortoluzzi A.J., Stulzer H.K. (2014). HPMC as a potential enhancer of nimodipine biopharmaceutical properties via ball-milled solid dispersions. Carbohydr. Polym..

[21] Ghosh I., Snyder J., Vippagunta R., Alvine M., Vakil R., Tong W.-Q., Vippagunta S. (2011). Comparison of HPMC based polymers performance as carriers for manufacture of solid dispersions using the melt extruder. Int. J. Pharm..

[22] Lim H.-T., Balakrishnan P., Oh D.H., Joe K.H., Kim Y.R., Hwang D.H., Lee Y.-B., Yong C.S., Choi H.-G. (2010). Development of novel sibutramine base-loaded solid dispersion with gelatin and HPMC: Physicochemical characterization and pharmacokinetics in beagle dogs. Int. J. Pharm..

[23] Jelić D., Liavitskaya T., Vyazovkin S. (2019). Thermal stability of indomethacin increases with the amount of polyvinylpyrrolidone in solid dispersion. Thermochim. Acta.

[24] Ben Osman Y., Liavitskaya T., Vyazovkin S. (2018). Polyvinylpyrrolidone affects thermal stability of drugs in solid dispersions. Int. J. Pharm..

[25] Smikalla M.M., Urbanetz N.A. (2007). The influence of povidone K17 on the storage stability of solid dispersions of nimodipine and polyethylene glycol. Eur. J. Pharm. Biopharm..

[26] Tran T.T.D., Tran P.H.L., Lee B.J. (2009). Dissolution-modulating mechanism of alkalizers and polymers in a nanoemulsifying solid dispersion containing ionizable and poorly water-soluble drug. Eur. J. Pharm. Biopharm..

[27] Tran T.T.D., Tran P.H.L., Choi H.G., Han H.K., Lee B.J. (2010). The roles of acidifiers in solid dispersions and physical mixtures. Int. J. Pharm..

[28] Sarode A.L., Malekar S.A., Cote C., Worthen D.R. (2014). Hydroxypropyl cellulose stabilizes amorphous solid dispersions of the poorly water soluble drug felodipine. Carbohydr. Polym..

[29] Chavan R.B., Rathi S., Jyothi V.G.S.S., Shastri N.R. (2019). Cellulose based polymers in development of amorphous solid dispersions. Asian J. Pharm. Sci..

[30] Winslow C.J., Nichols B.L.B., Novo D.C., Mosquera-Giraldo L.I., Taylor L.S., Edgar K.J., Neilson A.P. (2018). Cellulose-based amorphous solid dispersions enhance rifapentine delivery characteristics in vitro. Carbohydr. Polym..

[31] Vojinović T., Medarević D., Vranić E., Potpara Z., Krstić M., Djuriš J., Ibrić S. (2018). Development of ternary solid dispersions with hydrophilic polymer and surface adsorbent for improving dissolution rate of carbamazepine. Saudi Pharm. J..

[32] Janssens S., de Armas H.N., Roberts C.J., Van den Mooter G. (2008). Characterization of Ternary Solid Dispersions of Itraconazole, PEG 6000, and HPMC 2910 E5. J. Pharm. Sci..

[33] Goddeeris C., Willems T., Van den Mooter G. (2008). Formulation of fast disintegrating tablets of ternary solid dispersions consisting of TPGS 1000 and HPMC 2910 or PVPVA 64 to improve the dissolution of the anti-HIV drug UC 781. Eur. J. Pharm. Sci..

[34] Kumar V., Mintoo M.J., Mondhe D.M., Bharate S.B., Vishwakarma R.A., Bharate S.S. (2019). Binary and ternary solid dispersions of an anticancer preclinical lead, IIIM-290: In vitro and in vivo studies. Int. J. Pharm..

[35] Pezzoli R., Lyons J.G., Gately N., Higginbotham C.L. (2019). Stability studies of hot-melt extruded ternary solid dispersions of poorly-water soluble indomethacin with poly(vinyl pyrrolidone-co-vinyl acetate) and polyethylene oxide. J. Drug Deliv. Sci. Technol..

[36] Meng F., Meckel J., Zhang F. (2017). Investigation of itraconazole ternary amorphous solid dispersions based on povidone and Carbopol. Eur. J. Pharm. Sci..

[37] Ziaee A., Albadarin A.B., Padrela L., Faucher A., O’Reilly E., Walker G. (2017). Spray drying ternary amorphous solid dispersions of ibuprofen—An investigation into critical formulation and processing parameters. Eur. J. Pharm. Biopharm..

[38] Janssens S., Nagels S., De Armas H.N., D’Autry W., Van Schepdael A., Van den Mooter G. (2008). Formulation and characterization of ternary solid dispersions made up of Itraconazole and two excipients, TPGS 1000 and PVPVA 64, that were selected based on a supersaturation screening study. Eur. J. Pharm. Biopharm..

[39] Davis M.T., Potter C.B., Walker G.M. (2018). Downstream processing of a ternary amorphous solid dispersion: The impacts of spray drying and hot melt extrusion on powder flow, compression and dissolution. Int. J. Pharm..

[40] Li J., Liu P., Liu J.-P., Zhang W.-L., Yang J.-K., Fan Y.-Q. (2012). Novel Tanshinone II A ternary solid dispersion pellets prepared by a single-step technique: In vitro and in vivo evaluation. Eur. J. Pharm. Biopharm..

[41] Davis M.T., Potter C.B., Mohammadpour M., Albadarin A.B., Walker G.M. (2017). Design of spray dried ternary solid dispersions comprising itraconazole, soluplus and HPMCP: Effect of constituent compositions. Int. J. Pharm..

[42] Lu Y., Chen J., Yi S., Xiong S. (2019). Enhanced felodipine dissolution from high drug loading amorphous solid dispersions with PVP/VA and sodium dodecyl sulfate. J. Drug Deliv. Sci. Technol..

[43] Knopp M.M., Nguyen J.H., Becker C., Francke N.M., Jørgensen E.B., Holm P., Holm R., Mu H., Rades T., Langguth P. (2016). Influence of polymer molecular weight on in vitro dissolution behavior and in vivo performance of celecoxib:PVP amorphous solid dispersions. Eur. J. Pharm. Biopharm..

[44] Wang B., Wang D., Zhao S., Huang X., Zhang J., Lv Y., Liu X., Lv G., Ma X. (2017). Evaluate the ability of PVP to inhibit crystallization of amorphous solid dispersions by density functional theory and experimental verify. Eur. J. Pharm. Sci..

[45] Ziaee A., O’Dea S., Howard-Hildige A., Padrela L., Potter C., Iqbal J., Albadarin A.B., Walker G., O’Reilly E.J. (2019). Amorphous solid dispersion of ibuprofen: A comparative study on the effect of solution based techniques. Int. J. Pharm..

[46] Li J., Fan N., Li C., Wang J., Li S., He Z. (2017). The tracking of interfacial interaction of amorphous solid dispersions formed by water-soluble polymer and nitrendipine. Appl. Surf. Sci..

[47] Li J., Fan N., Wang X., Li C., Sun M., Wang J., Fu Q., He Z. (2017). Interfacial interaction track of amorphous solid dispersions established by water-soluble polymer and indometacin. Eur. J. Pharm. Sci..

[48] Hurley D., Carter D., Foong Ng L.Y., Davis M., Walker G.M., Lyons J.G., Higginbotham C.L. (2019). An investigation of the inter-molecular interaction, solid-state properties and dissolution properties of mixed copovidone hot-melt extruded solid dispersions. J. Drug Deliv. Sci. Technol..

[49] Tran T.T.D., Tran P.H.L., Lim J., Park J.B., Choi S.K., Lee B.J. (2010). Physicochemical principles of controlled release solid dispersion containing a poorly water-soluble drug. Ther. Deliv..

[50] Phuong H.L.T., Wei D., Beom-Jin L., Thao T.D.T. (2018). Current Designs of Polymer Blends in Solid Dispersions for Improving Drug Bioavailability. Curr. Drug Metab..

[51] Tran P.H.L., Tran T.T.D. (2020). Dosage form designs for the controlled drug release of solid dispersions. Int. J. Pharm..

[52] Vasconcelos T., Sarmento B., Costa P. (2007). Solid dispersions as strategy to improve oral bioavailability of poor water soluble drugs. Drug Discov. Today.

[53] Han R., Xiong H., Ye Z., Yang Y., Huang T., Jing Q., Lu J., Pan H., Ren F., Ouyang D. (2019). Predicting physical stability of solid dispersions by machine learning techniques. J. Control. Release.

[54] Ma X., Huang S., Lowinger M.B., Liu X., Lu X., Su Y., Williams R.O. (2019). Influence of mechanical and thermal energy on nifedipine amorphous solid dispersions prepared by hot melt extrusion: Preparation and physical stability. Int. J. Pharm..

[55] Jang S.W., Kang M.J. (2014). Improved oral absorption and chemical stability of everolimus via preparation of solid dispersion using solvent wetting technique. Int. J. Pharm..

[56] Li B., Konecke S., Wegiel L.A., Taylor L.S., Edgar K.J. (2013). Both solubility and chemical stability of curcumin are enhanced by solid dispersion in cellulose derivative matrices. Carbohydr. Polym..

[57] Vasconcelos T., Marques S., das Neves J., Sarmento B. (2016). Amorphous solid dispersions: Rational selection of a manufacturing process. Adv. Drug Deliv. Rev..

[58] Pandi P., Bulusu R., Kommineni N., Khan W., Singh M. (2020). Amorphous solid dispersions: An update for preparation, characterization, mechanism on bioavailability, stability, regulatory considerations and marketed products. Int. J. Pharm..

[59] Ma X., Williams R.O. (2019). Characterization of amorphous solid dispersions: An update. J. Drug Deliv. Sci. Technol..

[60] Beten D.B., Gelbcke M., Diallo B., Moës A.J. (1992). Interaction between dipyridamole and Eudragit S. Int. J. Pharm..

[61] Guo S., Wang G., Wu T., Bai F., Xu J., Zhang X. (2017). Solid dispersion of berberine hydrochloride and Eudragit^®^ S100: Formulation, physicochemical characterization and cytotoxicity evaluation. J. Drug Deliv. Sci. Technol..

[62] Monschke M., Wagner K.G. (2019). Amorphous solid dispersions of weak bases with pH-dependent soluble polymers to overcome limited bioavailability due to gastric pH variability—An in-vitro approach. Int. J. Pharm..

[63] Overhoff K.A., Moreno A., Miller D.A., Johnston K.P., Williams R.O. (2007). Solid dispersions of itraconazole and enteric polymers made by ultra-rapid freezing. Int. J. Pharm..

[64] Mayersohn M. (1990). Principles of drug absorption. Modern Pharmaceutics.

[65] Sherwood L. (2015). Human Physiology: From Cells to Systems.

[66] Maniruzzaman M., Morgan D.J., Mendham A.P., Pang J., Snowden M.J., Douroumis D. (2013). Drug–polymer intermolecular interactions in hot-melt extruded solid dispersions. Int. J. Pharm..

[67] Maniruzzaman M., Boateng J.S., Bonnefille M., Aranyos A., Mitchell J.C., Douroumis D. (2012). Taste masking of paracetamol by hot-melt extrusion: An in vitro and in vivo evaluation. Eur. J. Pharm. Biopharm..

[68] Phuong H.L.T., Wei D., Beom-Jin L., Thao T.D.T. (2019). Modulation of Drug Crystallization and Molecular Interactions by Additives in Solid Dispersions for Improving Drug Bioavailability. Curr. Pharm. Des..

[69] Sarode A.L., Sandhu H., Shah N., Malick W., Zia H. (2013). Hot melt extrusion (HME) for amorphous solid dispersions: Predictive tools for processing and impact of drug–polymer interactions on supersaturation. Eur. J. Pharm. Sci..

[70] Mathers A., Hassouna F., Malinová L., Merna J., Růžička K., Fulem M. (2019). Impact of Hot-Melt Extrusion Processing Conditions on Physicochemical Properties of Amorphous Solid Dispersions Containing Thermally Labile Acrylic Copolymer. J. Pharm. Sci..

[71] Parikh T., Gupta S.S., Meena A., Serajuddin A.T. (2016). Investigation of thermal and viscoelastic properties of polymers relevant to hot melt extrusion-III: Polymethacrylates and polymethacrylic acid based polymers. J. Excip. Food Chem..

[72] Hasegawa A., Kawamura R.I.E., Nakagawa H., Sugimoto I. (1985). Physical Properties of Solid Dispersions of Poorly Water-Soluble Drugs with Enteric Coating Agents. Chem. Pharm. Bull..

[73] Sotthivirat S., McKelvey C., Moser J., Rege B., Xu W., Zhang D. (2013). Development of amorphous solid dispersion formulations of a poorly water-soluble drug, MK-0364. Int. J. Pharm..

[74] Zhang Q., Zhao Y., Zhao Y., Ding Z., Fan Z., Zhang H., Liu M., Wang Z., Han J. (2018). Effect of HPMCAS on recrystallization inhibition of nimodipine solid dispersions prepared by hot-melt extrusion and dissolution enhancement of nimodipine tablets. Colloids Surf. B Biointerfaces.

[75] Vo A.Q., Feng X., Zhang J., Zhang F., Repka M.A. (2018). Dual mechanism of microenvironmental pH modulation and foam melt extrusion to enhance performance of HPMCAS based amorphous solid dispersion. Int. J. Pharm..

[76] Solanki N.G., Lam K., Tahsin M., Gumaste S.G., Shah A.V., Serajuddin A.T.M. (2019). Effects of Surfactants on Itraconazole-HPMCAS Solid Dispersion Prepared by Hot-Melt Extrusion I: Miscibility and Drug Release. J. Pharm. Sci..

[77] Higashi K., Hayashi H., Yamamoto K., Moribe K. (2015). The effect of drug and EUDRAGIT^®^ S 100 miscibility in solid dispersions on the drug and polymer dissolution rate. Int. J. Pharm..

[78] Song Y., Zemlyanov D., Chen X., Su Z., Nie H., Lubach J.W., Smith D., Byrn S., Pinal R. (2016). Acid-base interactions in amorphous solid dispersions of lumefantrine prepared by spray-drying and hot-melt extrusion using X-ray photoelectron spectroscopy. Int. J. Pharm..

[79] Shah N., Sandhu H., Phuapradit W., Pinal R., Iyer R., Albano A., Chatterji A., Anand S., Choi D.S., Tang K. (2012). Development of novel microprecipitated bulk powder (MBP) technology for manufacturing stable amorphous formulations of poorly soluble drugs. Int. J. Pharm..

[80] Hu Q., Choi D.S., Chokshi H., Shah N., Sandhu H. (2013). Highly efficient miniaturized coprecipitation screening (MiCoS) for amorphous solid dispersion formulation development. Int. J. Pharm..

[81] Shah N., Iyer R.M., Mair H.-J., Choi D., Tian H., Diodone R., Fahnrich K., Pabst-Ravot A., Tang K., Scheubel E. (2013). Improved Human Bioavailability of Vemurafenib, a Practically Insoluble Drug, Using an Amorphous Polymer-Stabilized Solid Dispersion Prepared by a Solvent-Controlled Coprecipitation Process. J. Pharm. Sci..

[82] Xie T., Taylor L.S. (2016). Dissolution performance of high drug loading celecoxib amorphous solid dispersions formulated with polymer combinations. Pharm. Res..

[83] Ngo H.V., Tran P.H.L., Lee B.J., Tran T.T.D. (2019). The roles of a surfactant in zein-HPMC blend solid dispersions for improving drug delivery. Int. J. Pharm..

[84] Six K., Verreck G., Peeters J., Brewster M., Van den Mooter G. (2004). Increased physical stability and improved dissolution properties of itraconazole, a class II drug, by solid dispersions that combine fast-and slow-dissolving polymers. J. Pharm. Sci..

[85] Thao T.D.T., Phuong H.L.T. (2017). Perspectives on Strategies Using Swellable Polymers in Solid Dispersions for Controlled Drug Release. Curr. Pharm. Des..

[86] Ohyagi N., Ueda K., Higashi K., Yamamoto K., Kawakami K., Moribe K. (2017). Synergetic Role of Hypromellose and Methacrylic Acid Copolymer in the Dissolution Improvement of Amorphous Solid Dispersions. J. Pharm. Sci..

[87] Marks J.A., Wegiel L.A., Taylor L.S., Edgar K.J. (2014). Pairwise Polymer Blends for Oral Drug Delivery. J. Pharm. Sci..

[88] Duarte Í., Corvo M.L., Serôdio P., Vicente J., Pinto J.F., Temtem M. (2016). Production of nano-solid dispersions using a novel solvent-controlled precipitation process—Benchmarking their in vivo performance with an amorphous micro-sized solid dispersion produced by spray drying. Eur. J. Pharm. Sci..

[89] Balogh A., Farkas B., Domokos A., Farkas A., Démuth B., Borbás E., Nagy B., Marosi G., Nagy Z.K. (2017). Controlled-release solid dispersions of Eudragit^®^ FS 100 and poorly soluble spironolactone prepared by electrospinning and melt extrusion. Eur. Polym. J..

[90] Balogh A., Farkas B., Pálvölgyi Á., Domokos A., Démuth B., Marosi G., Nagy Z.K. (2017). Novel Alternating Current Electrospinning of Hydroxypropylmethylcellulose Acetate Succinate (HPMCAS) Nanofibers for Dissolution Enhancement: The Importance of Solution Conductivity. J. Pharm. Sci..

[91] Kennedy M., Hu J., Gao P., Li L., Ali-Reynolds A., Chal B., Gupta V., Ma C., Mahajan N., Akrami A. (2008). Enhanced Bioavailability of a Poorly Soluble VR1 Antagonist Using an Amorphous Solid Dispersion Approach: A Case Study. Mol. Pharm..

[92] Sun D.D., Lee P.I. (2015). Probing the mechanisms of drug release from amorphous solid dispersions in medium-soluble and medium-insoluble carriers. J. Control. Release.

[93] Smeets A., Koekoekx R., Clasen C., Van den Mooter G. (2018). Amorphous solid dispersions of darunavir: Comparison between spray drying and electrospraying. Eur. J. Pharm. Biopharm..

[94] Hassouna F., Abo El Dahab M., Fulem M., De Lima Haiek A., Laachachi A., Kopecký D., Šoóš M. (2019). Multi-scale analysis of amorphous solid dispersions prepared by freeze drying of ibuprofen loaded acrylic polymer nanoparticles. J. Drug Deliv. Sci. Technol..

[95] Pignatello R., Amico D., Chiechio S., Spadaro C., Puglisi G., Giunchedi P. (2001). Preparation and Analgesic Activity of Eudragit RS100^®^ Microparticles Containing Diflunisal. Drug Deliv..

[96] Chen D., Tsay R.-J., Lin H.-I., Chen H., Chao S.-C., Ku H. (2000). Stabilization and sustained-release effect of Misoprostol with Methacrylate copolymer. Int. J. Pharm..

[97] Barzegar-Jalali M., Alaei-Beirami M., Javadzadeh Y., Mohammadi G., Hamidi A., Andalib S., Adibkia K. (2012). Comparison of physicochemical characteristics and drug release of diclofenac sodium–eudragit^®^ RS100 nanoparticles and solid dispersions. Powder Technol..

[98] Aceves J.M., Cruz R., Hernandez E. (2000). Preparation and characterization of Furosemide-Eudragit controlled release systems. Int. J. Pharm..

[99] Wiranidchapong C., Tucker I.G., Rades T., Kulvanich P. (2008). Miscibility and interactions between 17β-estradiol and Eudragit^®^ RS in solid dispersion. J. Pharm. Sci..

[100] Pignatello R., Ferro M., De Guidi G., Salemi G., Vandelli M.A., Guccione S., Geppi M., Forte C., Puglisi G. (2001). Preparation, characterisation and photosensitivity studies of solid dispersions of diflunisal and Eudragit RS100^®^ and RL100^®^. Int. J. Pharm..

[101] Albarahmieh E.A., Qi S., Craig D.Q.M. (2016). Hot melt extruded transdermal films based on amorphous solid dispersions in Eudragit RS PO: The inclusion of hydrophilic additives to develop moisture-activated release systems. Int. J. Pharm..

[102] Verma V., Sharma P., Sharma J., Kaur Lamba A., Lamba H.S. (2017). Development, characterization and solubility study of solid dispersion of Quercetin by solvent evaporation method. Mater. Today: Proc..

[103] Liu H., Kar N., Edgar K.J. (2012). Direct synthesis of cellulose adipate derivatives using adipic anhydride. Cellulose.

[104] Ngo H., Nguyen P.K., Van Vo T., Duan W., Tran V.T., Tran P.H.L., Tran T.T.D. (2016). Hydrophilic-hydrophobic polymer blend for modulation of crystalline changes and molecular interactions in solid dispersion. Int. J. Pharm..

[105] Yang M.-S., Cui F.-D., You B.-G., Fan Y.-L., Wang L., Yue P., Yang H. (2003). Preparation of sustained-release nitrendipine microspheres with Eudragit RS and Aerosil using quasi-emulsion solvent diffusion method. Int. J. Pharm..

[106] Huang J., Wigent R.J., Schwartz J.B. (2006). Nifedipine Molecular Dispersion in Microparticles of Ammonio Methacrylate Copolymer and Ethylcellulose Binary Blends for Controlled Drug Delivery: Effect of Matrix Composition. Drug Dev. Ind. Pharm..

[107] Huang J., Wigent R.J., Bentzley C.M., Schwartz J.B. (2006). Nifedipine solid dispersion in microparticles of ammonio methacrylate copolymer and ethylcellulose binary blend for controlled drug delivery: Effect of drug loading on release kinetics. Int. J. Pharm..

[108] Orienti I., Bigucci F., Luppi B., Cerchiara T., Zuccari G., Giunchedi P., Zecchi V. (2002). Polyvinylalcohol substituted with triethyleneglycolmonoethylether as a new material for preparation of solid dispersions of hydrophobic drugs. Eur. J. Pharm. Biopharm..

[109] Ueda K., Higashi K., Yamamoto K., Moribe K. (2014). The effect of HPMCAS functional groups on drug crystallization from the supersaturated state and dissolution improvement. Int. J. Pharm..

[110] Dinh H.T.T., Tran P.H.L., Duan W., Lee B.-J., Tran T.T.D. (2017). Nano-sized solid dispersions based on hydrophobic-hydrophilic conjugates for dissolution enhancement of poorly water-soluble drugs. Int. J. Pharm..

[111] Tran C.T.M., Tran P.H.L., Tran T.T.D. (2019). pH-independent dissolution enhancement for multiple poorly water-soluble drugs by nano-sized solid dispersions based on hydrophobic–hydrophilic conjugates. Drug Dev. Ind. Pharm..

[112] Lovrecich M., Nobile F., Rubessa F., Zingone G. (1996). Effect of ageing on the release of indomethacin from solid dispersions with Eudragits. Int. J. Pharm..

[113] Wendelboe J., Knopp M.M., Khan F., Chourak N., Rades T., Holm R. (2017). Importance of in vitro dissolution conditions for the in vivo predictability of an amorphous solid dispersion containing a pH-sensitive carrier. Int. J. Pharm..

